# Sound-Evoked Responses of Distinct Neuron Classes from the Tail of the Striatum

**DOI:** 10.1523/ENEURO.0201-22.2022

**Published:** 2022-09-29

**Authors:** Matthew B. Nardoci, Anna A. Lakunina, Devin C. Henderling, Jewlyssa C. Pedregon, Jennifer L. Mohn, Santiago Jaramillo

**Affiliations:** 1 Institute of Neuroscience, University of Oregon, Eugene, Oregon 97403; 2 Department of Biology, University of Oregon, Eugene, Oregon 97403; 3 Department of Human Physiology, University of Oregon, Eugene, Oregon 97403

**Keywords:** auditory, direct pathway, dopamine receptor D_1_, posterior striatum, subcortical

## Abstract

Given its inputs from auditory structures and neuromodulatory systems, the posterior tail of the striatum is ideally positioned to influence behavioral responses to acoustic stimuli according to context and previous rewards. Results from previous studies indicate that neurons in this striatal region display selective responses to sounds. However, it is not clear whether different striatal cell classes code for distinct features of sounds or how different striatal output pathways may use acoustic information to guide behavior. Here we compared the sound-evoked responses of posterior striatal neurons that form the striatal direct pathway (and express the dopamine receptor D_1_) to the responses of neighboring neurons in naive mice. We achieved this via optogenetic photo-identification of D_1_-expressing neurons during extracellular electrophysiological recordings in awake head-fixed mice of both sexes. We found that the frequency tuning of sound-responsive direct-pathway striatal neurons is comparable with that of their sound-responsive neighbors. Moreover, we found that both populations encode amplitude-modulated sounds in a similar fashion. These results suggest that different classes of neurons in the posterior striatum of naive animals have similar access to acoustic features conveyed by the auditory system even outside the context of an auditory task.

## Significance Statement

Sound-driven decision-making is a key component of the behavioral repertoire of an animal in their quest for positive outcomes. Subsets of neurons in the striatum (a brain area associated with motor control and the integration of reward information) receive inputs from the auditory system, yet what features of sounds are accessible to specific striatal cell classes is not well understood. We found that multiple classes of posterior striatal neurons have access to detailed spectrotemporal acoustic features and could therefore potentially influence behavioral responses according to these properties of sounds.

## Introduction

The striatum, as the primary input structure of the basal ganglia and a target of extensive dopaminergic inputs, is ideally positioned to influence behavioral responses to sensory stimuli according to context and previous rewards. Neurons in the posterior tail of the striatum receive numerous inputs from the auditory thalamus and the auditory cortex (Hintiryan et al., 2016; Chen et al., 2019; Ponvert and Jaramillo, 2019; Valjent and Gangarossa, 2021), and these striatal neurons display robust responses to sounds (Bordi and LeDoux, 1992; Znamenskiy and Zador, 2013; Zhong et al., 2014; Guo et al., 2018). It is not known, however, whether different striatal cell classes code for distinct features of sounds or how different striatal output pathways may use acoustic information to guide behavior. A key step toward understanding the processing of sounds by striatal circuits and the role of different striatal cells in auditory learning is the characterization of sound-evoked responses by distinct striatal neuron classes in naive animals.

The striatum, including the posterior tail portion, is composed of a range of neuron classes with different gene expression and physiological profiles, including fast-spiking parvalbumin-expressing interneurons, spontaneously active cholinergic interneurons, and the abundant principal projection neurons (Kawaguchi, 1997; Valjent and Gangarossa, 2021). The large majority of these cells consists of medium spiny neurons that form the two main outputs of the striatum: the direct (striatonigral) pathway, composed of cells that express the dopamine receptor D_1_; and the indirect (striatopallidal) pathway, with cells that express the dopamine receptor D_2_ (Gerfen et al., 1990; Kreitzer and Malenka, 2008). Anatomical data suggest that, for some striatal regions, cortical sensory neurons differentially innervate each of these striatal pathways (Lei et al., 2004; Wall et al., 2013). Moreover, excitatory synapses onto direct and indirect pathway neurons exhibit different synaptic transmission and plasticity properties (Kreitzer and Malenka, 2007). Together, these observations raise the possibility that neurons from different classes in the posterior striatum are differentially influenced by sensory signals.

Using optogenetic photo-identification of specific cell populations in the posterior tail of the striatum of naive mice, we characterized the sound-evoked responses of neurons that express the dopamine receptor D_1_ and compared these responses with those from neighboring neurons. We found that on average, sound-responsive D_1_-expressing posterior striatal neurons have similar frequency tuning to their non-D_1_ sound-responsive neighbors, and that both populations represent amplitude-modulated noise in a similar fashion. These results suggest that different classes on neurons in the posterior striatum, likely including both major output pathways, have access to acoustic features conveyed from the auditory system in naive animals even outside the context of an auditory task.

## Materials and Methods

### Animals

A total of 15 transgenic adult DRD1::ChR2 mice of both sexes, were used in this study. This mouse line was generated by crossing animals that express Cre recombinase in neurons positive for the dopamine receptor D_1_ (MMRRC; RRID:MMRRC_036916-UCD) with mice that express the light-gated ion channel channelrhodopsin-2 (ChR2) in a Cre-dependent manner (stock #012569, The Jackson Laboratory). All procedures were conducted in accordance with the National Institutes of Health standards and were approved by the Institutional Animal Care and Use Committee of the University of Oregon.

### Auditory stimuli

Experiments were performed inside a single-walled sound-isolation box (IAC Acoustics). Auditory stimuli were presented in an open-field configuration from a speaker (model MF1, Tucker-Davis Technologies) contralateral to the side of electrophysiological recordings. Speakers were calibrated using an ultrasonic microphone (model ANL-940–1, Med Associates) to obtain the desired sound intensity level for frequencies between 1 and 40 kHz. Stimuli were generated using Python software developed in-house (https://taskontrol.readthedocs.io/). The ensemble of auditory stimuli for evaluating frequency tuning consisted of pure-tone pips (duration, 100 ms) at 16 frequencies logarithmically spaced between 2 and 40 kHz and at 11 different intensities (15–70 dB SPL in 5 dB steps). We presented at least nine repetitions per frequency–intensity combination with interstimulus intervals randomized in the range 0.7–0.9 s. Stimuli for evaluating responses to temporal sound features were sinusoidally amplitude-modulated white noise at 11 modulation rates logarithmically spaced between 4 and 128 Hz (modulation depth, 100%; duration, 500 ms; 60 dB SPL maximum). We presented at least 20 repetitions per condition using an interstimulus interval randomized in the range 0.9–1.1 s. All stimuli had a 2 ms ramp-up and ramp-down. During sound presentation, mice were awake and head fixed on top of a freely moving wheel, leaving them free to move their limbs while their heads remained stationary.

### Surgical procedure

Mice were surgically implanted with a head bar to allow for head-fixed recordings. Animals were anesthetized with isoflurane through a nose cone on a stereotaxic apparatus. Bilateral craniotomies (AP, −1 to −2 mm from bregma; ML, ±2.9 to 4 mm from midline) and durotomies were performed to allow for acute recordings from the most posterior region of the dorsal striatum. Plastic wells were attached around each craniotomy and filled with a silicone elastomer (Sylgard 170, Dow Corning) to protect the surface of the brain and retain moisture when not recording. All animals were monitored after surgery and recovered fully before electrophysiological experiments.

### Electrophysiological recordings and optogenetic stimulation

Animals were habituated to the sound presentation and the head-fixed apparatus for at least 1 h, 1 d before the first day of recording. Electrical signals were collected with an acquisition system (catalog # RHD2000, Intan Technologies) and Open Ephys software (www.open-ephys.org), using 32-channel silicon probes with electrodes arranged as tetrodes (A 4 × 2-tet configuration; NeuroNexus). The shanks of the probes were marked with a fluorescent dye (DiI, catalog #V22885; or DiD catalog #V22887, Thermo Fisher Scientific) before penetration of the brain to assist in the identification of shank location postmortem. Before neural recordings, animals were head fixed, the silicone elastomer was removed, and the electrodes were inserted through the craniotomy. The probe was held in a vertical position and lowered 2.9 mm from the brain surface. We waited for at least 15 min for the probe to settle before initiating recordings. Neural recordings were performed at multiple depths on each penetration, with recording sites typically 100–150 *μ*m apart to avoid recording from the same cells twice. Multiple penetrations were performed for each animal. A few recording sessions included only the presentation of one stimulus type [pure tones or amplitude-modulated (AM) noise], while the large majority of recording sessions included both.

A 50-*μ*m-core diameter Polymicro optical fiber (part #1068001596, Molex) was attached to the silicon probe between the middle shanks and ∼200 *μ*m above the top tetrode. The optical fiber was connected to a 445 nm laser calibrated to deliver 2 mW at the fiber tip. To identify D_1_-expressing neurons, we ran at least 50 laser stimulation trials before the sound presentation trials.

### Estimation of recording location

At the conclusion of the experiments, animals were deeply anesthetized with euthasol and perfused through the heart with 4% paraformaldehyde (PFA). Brains were extracted and left in 4% paraformaldehyde for at least 24 h before slicing. Brain slices (thickness, 50 or 100 *μ*m) were prepared under PBS using a vibratome (model VT1000 S, Leica) and imaged using a fluorescence microscope (Axio Imager 2, Carl Zeiss) with a 1.25× and 2.5× objective (numerical aperture, 0.16). To determine the locations of our recordings, we manually registered each brain slice containing dye fluorescence from a recording track to the corresponding coronal section in the Allen Mouse Common Coordinate Framework [Common Coordinate Framework version 3, 2015, Allen Institute for Brain Science; Allen Brain Atlas API (application programming interface), http://brain-map.org/api/index.html]. Recordings identified to be from the cerebral cortex were excluded from further analysis.

### Characterization of ChR2-enhanced yellow fluorescent protein expression

Expression of ChR2-enhanced yellow fluorescent protein (EYFP) in the striatum of experimental animals was verified during the estimation of recording locations. To further evaluate the expression of ChR2-EYFP in the posterior striatum of DRD1::ChR2 mice at cellular resolution, brain slices from two more mice were prepared for confocal imaging. Animals were deeply anesthetized with euthasol and transcardially perfused with 4% PFA. Brains were extracted and left in 4% PFA for 24 h, then cryoprotected in 30% sucrose for 48 h. The brains were then sectioned (20 *μ*m thick) using a cryostat (model CM3050 S, Leica), and brain slices (1.8–2 mm posterior from bregma) were mounted and DAPI stained. Images were acquired using a confocal laser-scanning microscope (model LSM 880, Zeiss).

### Data analysis

#### Spike sorting and selection of D_1_-expressing neurons

Spiking activity was detected by applying a threshold (40–45 *μ*V) to bandpass-filtered electrical signals (300–6000 Hz) measured by the electrodes. The activity from single units was isolated offline using the automated expectation maximization clustering algorithm Klustakwik (Kadir et al., 2014). Isolated clusters were only included in the analysis if <5% of interspike intervals were <2 ms. We also calculated a spike quality index, defined as the ratio between the peak amplitude of the spike waveform and the average variance, calculated using the channel with the largest amplitude. Cells were only included in the analysis if they had a spike quality index >2. The analysis also excluded clusters identified as having noisy spike waveforms from visual inspection. Last, only neurons that had a firing rate (either spontaneous or evoked) of at least 1 spike/s were included in the analysis. Cells with lower firing rates were excluded because we considered our measurements from these potential cells to be unreliable.

To identify D_1_-expressing neurons, the spike-sorting algorithm was applied to the combination of laser trials, trials with pure tones, and trials with AM sounds, such that each neuron could be identified by its spike shape across all stimulation conditions. Neurons were classified as D_1_-expressing if their onset response to laser stimulation (first 50 ms) was statistically larger (*p *<* *0.01, Wilcoxon signed-rank test) than the baseline firing estimated from 200 ms before stimulus onset. We chose the first 50 ms (of the 100 ms laser pulse) to minimize confounds resulting from network recurrence. Neurons were classified as non-D_1_ if the response to the laser was a suppression in firing or if the *p*-value associated with the laser response was >0.1. Neurons with positive responses to the laser and *p*-values between 0.01 and 0.1 were excluded from the analysis. Changing criteria slightly (e.g., requiring non-D_1_ neurons to have laser-evoked *p*-values >0.1) did not qualitatively affect the results. Additional comparisons between neuron classes were performed while restricting the non-D_1_ population to only those neurons that were recorded from sites where D_1_ cells were found. That is, if no D_1_ neurons were observed on a tetrode during a session, cells from that tetrode were not included.

#### Estimation of frequency tuning and responses to pure tones

To determine whether a neuron was responsive to pure tones, we tested whether the evoked response during sound presentation (0–100 ms) was statistically different from the baseline spontaneous firing (measured during the 200 ms before sound onset) for any of the frequencies presented, collapsed across intensities. Because this test was performed for each of the 16 frequencies presented, we performed a Bonferroni correction for multiple comparisons (resulting in *α* = 0.05/16 = 0.0031). A tone response index (TRI) was calculated for the sound frequency that elicited the largest change from baseline firing for each neuron: $\text{TRI}=(r_{e}-r_{b})/(r_{e}+r_{b})$, where *r_e_* is the average evoked response for that sound frequency and *r_b_* is the baseline spontaneous firing. To characterize the sound frequency tuning of each neuron, we first determined the frequency response area (FRA), defined as the set of frequency–intensity pairs for which the response of the cell was greater than a response threshold set to the baseline firing rate plus 20% of the difference between baseline and the maximum firing rate of the cell under any condition (Ponvert and Jaramillo, 2019). The characteristic frequency (CF) of a neuron was defined as the frequency with the lowest sound intensity inside the FRA where 85% of the intensities above were also within the FRA. Neurons were categorized as having a steep slope in the high-frequency flank of the FRA if this slope (calculated from the border of the FRA at maximum intensity and the CF) was >100 dB/octave.

The tuning bandwidth of each neuron was first estimated by fitting a Gaussian function to the average firing rate evoked by each frequency, collapsed across sound intensities, and measuring the full-width at half-maximum of this function (which for a Gaussian corresponds to 2.355*σ*, where *σ* is the SD). Only neurons with *R*^2^ values >0.01 were included in the comparisons of tuning bandwidth. For neurons with sufficiently low thresholds, we also estimated the bandwidth at 10 dB above threshold (BW10) and BW40. In these cases, the Gaussian function was fit to the responses at a particular intensity, and the method described above for estimating the full-width at half-maximum was applied.

To estimate differences in response dynamics to pure tones, we further analyzed neural responses from the same recording sessions used for estimating frequency tuning. We first estimated the latency of response of a neuron by pooling all trials with stimuli within its FRA, calculating a peristimulus time histogram (smoothed with a Hanning window 11 ms wide), and finding the time it took this signal to reach the midpoint between baseline and peak firing. This analysis was restricted to neurons with intensity thresholds <60 dB to get a sufficient number of trials, since the FRA of high-threshold neurons is generally very small. Neurons were also excluded if the automatic method yielded a negative response latency (e.g., because of a low firing rate). We also calculated an onset-to-sustained index (OSI) as $\text{OSI}=(r_{o}-r_{s})/(r_{o}+r_{s})$, where *r_o_* is the average firing rate during the early part of the stimulus (0–50 ms) and *r_s_* is the average firing rate during the late part of the stimulus (50–100 ms). This analysis focused on stimuli that fell within the FRA of each neuron.

#### Estimation of responses to AM sounds

To determine whether a neuron was responsive to AM noise, we tested whether the evoked response was statistically different from the baseline spontaneous firing (measured during the 200 ms before sound onset) for any of the AM rates presented. Because neurons in the auditory system often show substantially different responses at the onset versus the sustained periods of AM stimuli, we performed separate tests for each period: onset (0–100 ms) and sustained (100–500 ms). These tests were performed for each of the 11 AM rates presented; thus, we performed a Bonferroni correction for multiple comparisons (resulting in *α* = 0.05/11 = 0.0045). A sustained response index (SRI) was calculated for the AM rate that elicited the largest change from baseline firing for each neuron: $\text{SRI}=(r_{e}-r_{b})/(r_{e}+r_{b})$, where *r_e_* is the average evoked response for that AM rate during the sustained period and *r_b_* is the baseline spontaneous firing.

We estimated the rate modulation transfer function (Eggermont, 1994) for each responsive neuron by quantifying the average firing rate during the sustained response period (see Fig. 5*A–D*, middle panels, examples of transfer functions). AM rate selectivity was estimated by using an index that compared the maximum and minimum evoked firing rates during the sustained period across AM rates: $\left(r_{\text{max}}-r_{\text{min}})/(r_{\text{max}}+r_{\text{min}}\right)$. To evaluate how well the evoked spikes synchronized to the modulation of the stimulus, we used a vector strength metric on the sustained response for each cell (see Fig. 5*A–D*, right panels, examples). We then performed a Rayleigh’s test to determine the AM rates for which a neuron displayed statistically significant synchronization of its firing with respect to the phase of the amplitude modulation. This test included a Bonferroni correction for multiple comparisons (*α* = 0.0045). For each neuron, we estimated the highest AM rate where the firing of the neuron was significantly synchronized to the modulation period.

### Statistics

Throughout the study, we used nonparametric statistical tests implemented by the Python package SciPy (Virtanen et al., 2020). When comparing evoked firing rates to spontaneous rates (e.g., for responses to laser stimulation or sound stimulation), we used a test for related paired samples (Wilcoxon signed-rank test), where each trial provides one pair. When comparing measurements across two populations of cells, we used a nonparametric tests for two independent samples (Mann–Whitney *U* rank test). The comparisons associated with the histograms (see Figs. 3*I*, 5*E*) were performed using the absolute value of the response index in each case, while the triangles indicate the median values separately for positive and negative responses. Further details on the comparisons made and statistical tests used are presented in Table 1.

**Table 1:** Summary of statistical analyses

Figure	Measurement	Comparison	*N* cells	Statistical test	Results
	Fraction of sound-responsive cells	D_1_ cells vs non-D_1_ cells	D_1_ = 208 of 482 nD_1_ = 135 of 465	Fisher’s exact test	*p *<* *0.0001
	Fraction of sound-responsive cells	D_1_ cells vs site-restricted non-D_1_ cells	D_1_ = 208 of 482 nD_1_ = 79 of 197	Fisher's exact test	*p *=* *0.027
3*I*	Tone response index	D_1_ cells vs non-D_1_ cells	D_1_ = 400 nD_1_ = 376	Mann–Whitney *U* rank test	*p *=* *0.0006 *U *=* *85880
	Tone response index	D_1_ cells vs site-restricted non-D_1_ cells	D_1_ = 400 nD_1_ = 188	Mann–Whitney *U* rank test	*p *=* *0.156 *U *=* *34873
	Baseline firing	D_1_ cells vs non-D_1_ cells	D_1_ = 400 nD_1_ = 376	Mann–Whitney *U* rank test	*p *=* *0.496 *U *=* *73073
	Best frequency	D_1_ cells vs non-D_1_ cells	D_1_ = 115 nD_1_ = 79	Mann–Whitney *U* rank test	*p *=* *0.915 *U *=* *4583
	Characteristic frequency	D_1_ cells vs non-D_1_ cells	D_1_ = 115 nD_1_ = 79	Mann–Whitney *U* rank test	*p *=* *0.41 *U *=* *4852
3*J*	Intensity threshold	D_1_ cells vs non-D_1_ cells	D_1_ = 115 nD_1_ = 79	Mann–Whitney *U* rank test	*p *=* *0.0015 *U *=* *5754
3*K*	Frequency tuning bandwidth	D_1_ cells vs non-D_1_ cells	D_1_ = 115 nD_1_ = 79	Mann–Whitney *U* rank test	*p *=* *0.086 *U *=* *3883
	BW10	D_1_ cells vs non-D_1_ cells	D_1_ = 97 nD_1_ = 70	Mann–Whitney *U* rank test	*p *=* *0.058 *U *=* *3981
	BW40	D_1_ cells vs non-D_1_ cells	D_1_ = 18 nD_1_ = 24	Mann–Whitney *U* rank test	*p *=* *0.809 *U *=* *206
3*L*	Fraction of steep slope cells	D_1_ cells vs non-D_1_ cells	D_1_ = 17 of 75 nD_1_ = 21 of 73	Fisher's exact test	*p *=* *0.574
	Response latency	D_1_ cells vs non-D_1_ cells	D_1_ = 65 nD_1_ = 64	Mann–Whitney *U* rank test	*p *=* *0.656 *U *=* *1985
4*G*	Onset-to-sustain index	D_1_ cells vs non-D_1_ cells	D_1_ = 49 nD_1_ = 48	Mann–Whitney *U* rank test	*p *=* *0.256 *U *=* *1334
5*E*	AM sustained response index	D_1_ cells vs non-D_1_ cells	D_1_ = 475 nD_1_ = 460	Mann–Whitney *U* rank test	*p *=* *0.0013 *U *=* *121753
	AM sustained response index	D_1_ cells vs site-restricted non-D_1_ cells	D_1_ = 475 nD_1_ = 193	Mann–Whitney *U* rank test	*p *=* *0.212 *U *=* *42792
	Preferred AM rate	D_1_ cells vs non-D_1_ cells	D_1_ = 81 nD_1_ = 52	Mann–Whitney *U* rank test	*p *=* *0.744 *U *=* *2175
5*F*	AM rate selectivity	D_1_ cells vs non-D_1_ cells	D_1_ = 81 nD_1_ = 52	Mann–Whitney *U* rank test	*p *=* *0.578 *U *=* *2227
5*G*	Highest synchronization rate	D_1_ cells vs non-D_1_ cells	D_1_ = 53 nD_1_ = 30	Mann–Whitney *U* rank test	*p *=* *0.832 *U *=* *817

## Results

### Distinct classes of posterior striatal neurons respond to sound stimuli

**Figure 1.**
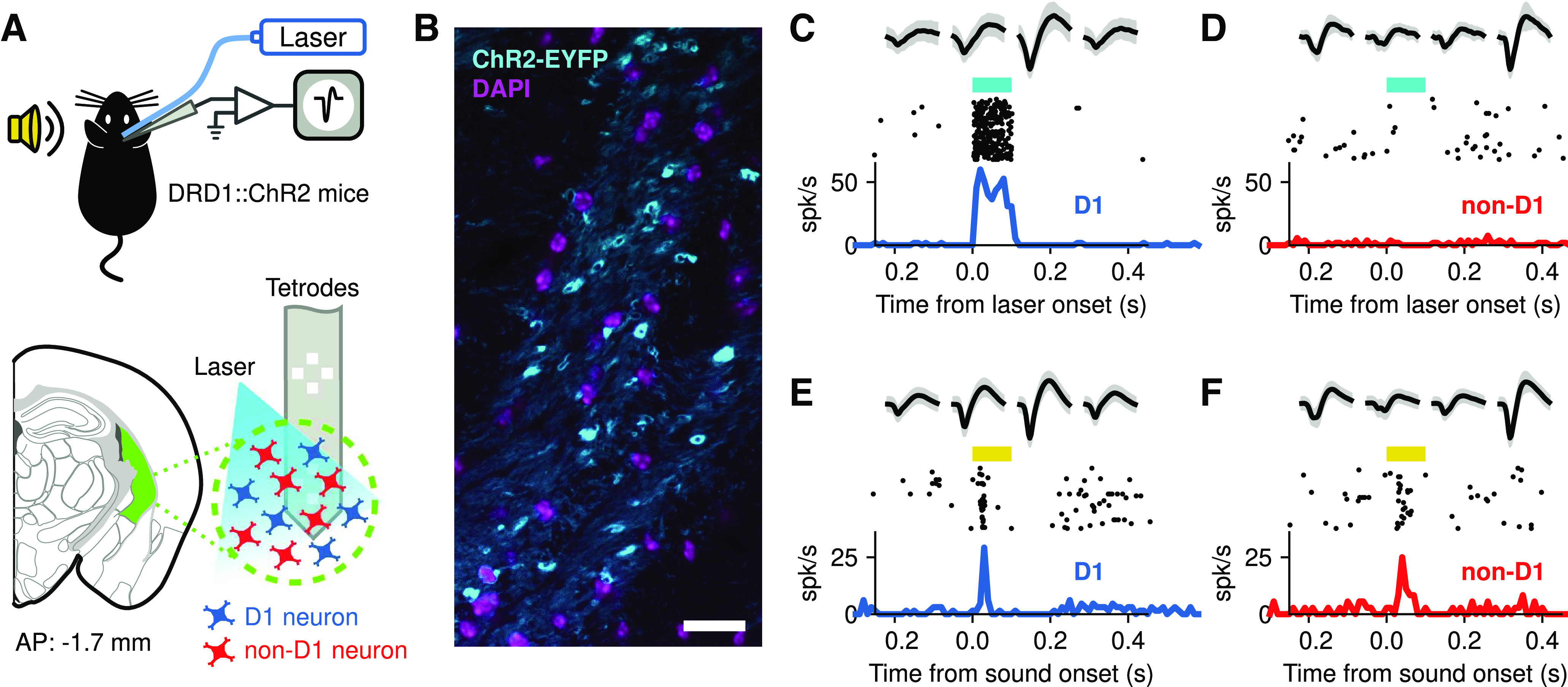
Photo-identification of D_1_-expressing striatal neurons. ***A***, Extracellular recordings of neurons from the posterior tail of the striatum (green area) of awake head-fixed DRD1::ChR2 mice during sound presentation. D_1_-expressing neurons were identified during electrophysiological recordings by evaluating neural responses to blue laser stimulation. ***B***, Expression of ChR2-EYFP in the posterior striatum of DRD1::ChR2 mice. Scale bar, 20 *μ*m. ***C***, Example responses to laser stimulation (cyan bar) from a striatal neuron. The top row shows the average (black) and SD (gray) of the spike shape collected from each channel of a tetrode in the silicon probe. The middle row shows the firing for each presentation of the laser, and the bottom row the peristimulus time histogram. The early and consistent response indicates that this cell expresses ChR2 and therefore is a D_1_ neuron. ***D***, Example of a cell that did not respond to the laser. Because of this, the cell is considered to be non-D_1_. ***E***, Firing of the cell in ***C*** evoked by a 9.9 kHz pure tone (yellow bar). Note that the spike shapes match those in ***B***. ***F***, Firing of the cell in ***D*** evoked by a 9.9 kHz pure tone. Spike shapes match those in ***D***.

To determine whether the representation of sounds differed across neuron classes in the posterior tail of the striatum, we recorded sound-evoked responses of photo-identified D_1_-expressing neurons and neighboring (non-D_1_) neurons in this brain region from naive awake mice using silicon multichannel probes that have electrodes organized as tetrodes (Fig. 1*A*). Photo-identification of D_1_ neurons during electrophysiological recordings was made possible by using DRD1::ChR2 mice, which express the light-gated ion channel ChR2 in D_1_ neurons (Fig. 1*B*), and evaluating the spiking responses of each recorded neuron to laser stimulation delivered via an optical fiber attached to the recording probe. This method for *in vivo* identification of a genetically defined neuronal populations has been extensively used and validated in several brain regions, including the striatum (Lima et al., 2009; Kravitz et al., 2013; Lakunina et al., 2020). Figure 1*C* shows an example striatal neuron that responds reliably to laser stimulation; the quick strong response after laser onset indicates that this neuron expresses D_1_. This neuron also shows reliable responses to sound, a pure tone in this case (Fig. 1*E*). In the same brain region, we found neurons that showed no response to laser stimulation (Fig. 1*D*), but reliably respond to sound (Fig. 1*F*).

**Figure 2.**
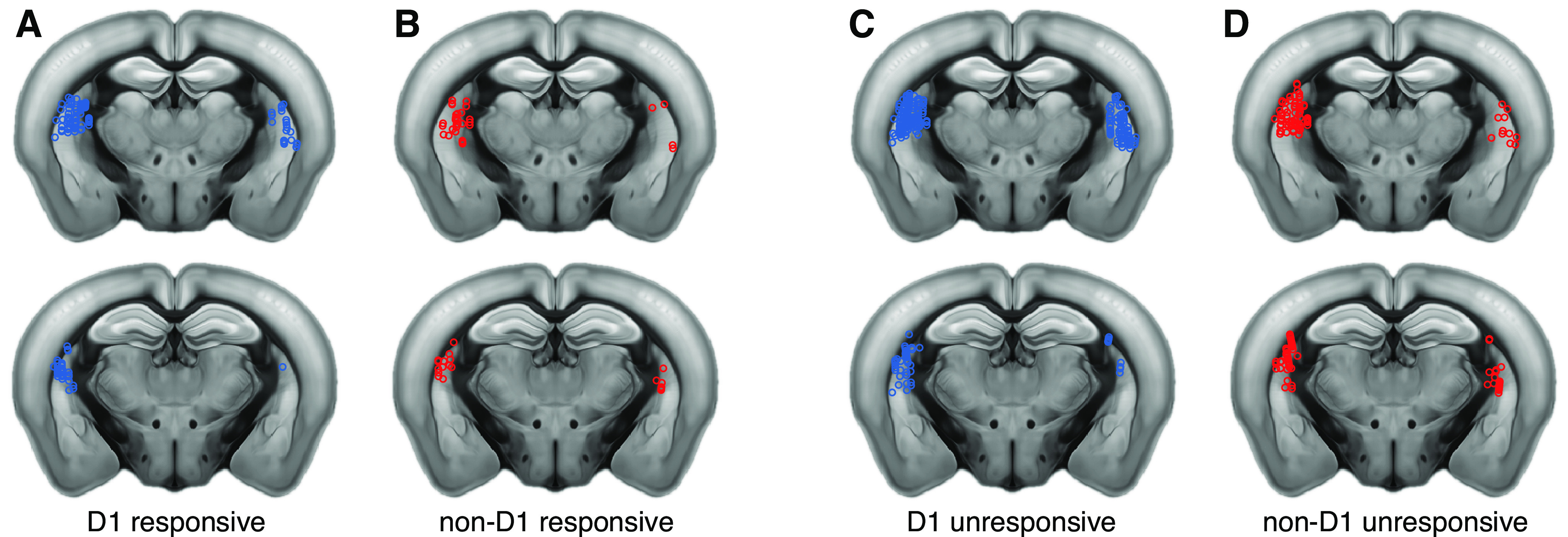
Location of recorded striatal neurons. ***A***, Coronal slices showing sites in both hemispheres, where we found D_1_ neurons that responded to at least one of the sounds in our ensemble (a combination of pure tones and amplitude modulated noise). Each circle represents one recording site. Sites are collapsed onto one of two anteroposterior locations shown: approximately −1.35 mm (top) and −1.75 mm (bottom) from bregma. The ventral region of the posterior tail of the striatum was not sampled in our experiments. ***B***, Coronal slices as in ***A*** showing sites where we found sound-responsive non-D_1_ neurons. ***C***, Sites where we found D_1_ neurons that did not respond to sounds. ***D***, Sites where we found non-D_1_ neurons that did not respond to sounds.

From our sample of cells, we identified 482 neurons as having statistically significant positive laser-evoked responses (Wilcoxon signed-rank test, *p *<* *0.01) and therefore classified as D_1_-expressing, and 465 classified as non-D_1_ neurons. From these populations, we found that 43% of D_1_ neurons and 29% of non-D_1_ neurons showed a reliable evoked response to at least one sound in our stimulus ensemble, a mix of pure tones of different frequencies and amplitude-modulated noise at different modulation rates. This difference in the fraction of responsive cells from each class was statistically significant (Fisher’s exact test, *p *<* *0.0001), although it was much less apparent when the analysis was restricted to the 197 non-D_1_ cells that were recorded from sites where D_1_ cells were found (43% D_1_ vs 40% non-D_1_ sound-responsive neurons; Fisher’s exact test, *p *=* *0.027). Figure 2 shows the estimated recording locations where we found sound-responsive neurons of each type, compared with recording locations where neurons were not sound responsive. There were no major differences between the locations of responsive and unresponsive neurons.

The observations described above indicate that subsets of medium spiny neurons in the posterior tail of the striatum that express the dopamine receptor D_1_, as well as neighboring non-D_1_ neurons, display reliable responses to sounds. We next wanted to test whether the sound-evoked responses and acoustic features encoded by these neurons differ between the two populations.

### D_1_-expressing neurons and their neighbors display similar sound frequency tuning

**Figure 3.**
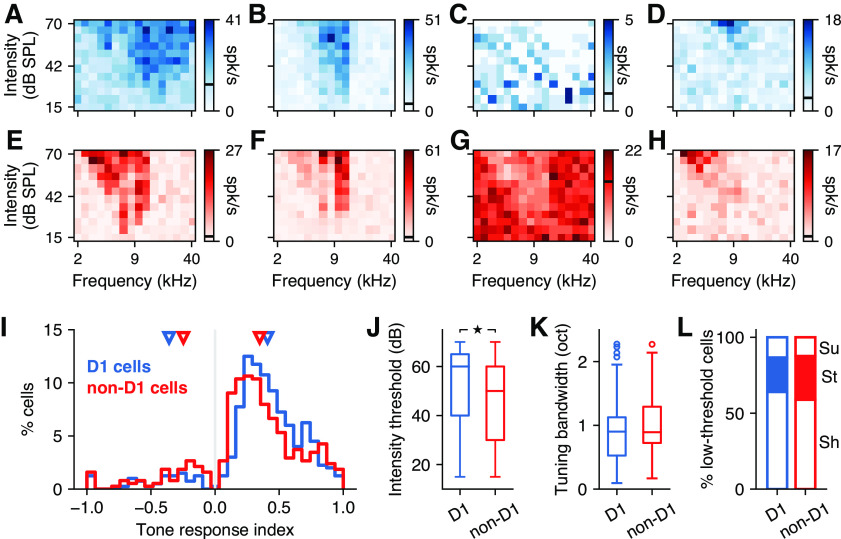
D_1_-expressing striatal neurons have comparable sound frequency tuning to neighboring neurons. ***A***, Example frequency–intensity tuning curve from a D_1_ striatal neuron in response to 100 ms pure tones. The spontaneous firing rate is shown as a black line in the scale bar. ***B***, D_1_ neuron that has a much steeper slope at the high-frequency flank of the response area. ***C***, D_1_ neuron that responded to sounds by decreasing its firing. ***D***, D_1_ neuron with a very high-intensity threshold. ***E*–*H***, Same as ***A–D*** for non-D_1_ striatal neurons. ***I***, Tone-evoked response index for the tone frequency that elicited the largest response in each neuron of each class. Triangles indicate the median response index calculated separately for neurons that responded by increasing or decreasing their firing. On average, D_1_ neurons showed larger responses than non-D_1_ neurons (Mann–Whitney *U* rank test, *p *=* *0.0006). ***J***, Intensity thresholds were slightly higher for D_1_ neurons compared with non-D_1_ neurons (Mann–Whitney *U* rank test, *p *=* *0.001), but both cell types spanned the full range of possible thresholds. ***K***, Frequency tuning bandwidth was similar between D_1_ and non-D_1_ neurons (Mann–Whitney *U* rank test, *p *=* *0.086). ***L***, The proportions of low-threshold cells with each shape of frequency response area were similar between D_1_ and non-D_1_ neurons. Sh, Shallow high-frequency slope (as in ***A***); St, steep high-frequency slope (as in ***B***); Su, suppressed response (as in ***C***).

To test whether D_1_ neurons in the posterior striatum encoded the frequency of sounds with different fidelity compared with other neurons in this region, we evaluated the evoked responses of identified D_1_ and non-D_1_ cells to pure tones of different frequencies and intensities. A total of 400 D_1_ and 376 non-D_1_ neurons were recorded during the presentation of pure tones. Figure 3*A–D* shows the average responses of example D_1_ neurons for each frequency–intensity combination, demonstrating a clear tuning to specific frequencies and a dependence on sound intensity for these cells. As has been observed in other regions of the auditory system (Egorova et al., 2001; Palmer et al., 2013), we found neurons with different shapes of tuning, characterized by the FRA. These included cells with a mostly symmetric FRA (Fig. 3*A*), cells with a steep slope in the high-frequency flank of the FRA (Fig. 3*B*), cells that responded by suppressing their firing (Fig. 3*C*), and cells with very high-intensity thresholds (Fig. 3*D*). All of these types of responses were also apparent in non-D_1_ neurons (Fig. 3*E–H*).

To evaluate differences between D_1_ and non-D_1_ neurons, we first calculated a tone response index that compared the evoked response to the baseline firing for each neuron and plotted this index (Fig. 3*I*) for the sound frequency that elicited the most reliable evoked response in each neuron (pooled across intensities). The distribution of these best frequencies (range: D_1_, 3.6–26.8 kHz; non-D_1_, 3–26.8 kHz) was similar between the two populations of neurons (Mann–Whitney *U* rank test, *p *=* *0.9). As expected, most neurons (even if they did not have a statistically significant response to sounds) had firing rates evoked by the best stimulus that differed from the spontaneous firing, and therefore show a response index different from zero (Fig. 3*I*). Positive values indicate that the evoked firing was larger than the spontaneous firing. Negative values, indicating a decrease in firing, were much less common (D_1_ cells, 41 of 400 = 10.2%; non-D_1_ cells, 59 of 376 = 15.7%). Cells that showed negative responses, as those illustrated in Figure 3, *C* and *G*, had on average higher spontaneous firing rates than those with positive responses for both D_1_ cells (median, 1.4 vs 0.88 spikes/s; Mann–Whitney *U* rank test, *p *=* *0.015) and non-D_1_ cells (median, 2.37 vs 0.77 spikes/s; Mann–Whitney *U* rank test, *p *<* *1*e* – 5), although there was no difference in spontaneous firing rate between D_1_ and non-D_1_ cells (Mann–Whitney *U* rank test, *p *=* *0.496).

Tone response index values largely overlapped across the two populations of cells, yet we found that the strongest sound-evoked changes in firing for D_1_ neurons were larger than those for non-D_1_ neurons (median absolute index: D_1_, 0.41 vs non-D_1_, 0.35; Mann–Whitney *U* rank test, *p *=* *0.0006). Moreover, the median values (Fig. 3*I*, triangles) were larger for D_1_ neurons that had positive evoked responses and smaller for neurons with negative evoked responses, compared with median values for non-D_1_ neurons. However, this difference disappeared when we restricted our analysis to the 188 non-D_1_ neurons recorded in sites (i.e., tetrodes) where D_1_ cells were also found (median: D_1_, 0.42 vs non-D_1_, 0.44; Mann–Whitney *U* rank test, *p *=* *0.156), suggesting that this result is influenced by the exact location of each recording site.

We next focused on the characteristics of the frequency response area of sound-responsive neurons. We found that the CF across neurons, which we observed in the range 3–22 kHz for D_1_ and 2.4–18 kHz for non-D_1_, was similar between the two populations (*p *=* *0.408, Mann–Whitney *U* rank test). We then tested whether neurons were tonotopically organized and observed a small but statistically significant correlation between the CF of neurons and their location in the medial–lateral axis, similar for both populations (D_1_: *r *=* *0.27, *p *=* *0.0021; non-D_1_: *r *=* *0.33, *p *=* *0.0059; Spearman’s correlation), with medial neurons having higher CFs. We found no organization in either the dorsoventral or anteroposterior axes. We also found that while neurons from both classes spanned the range of tested intensity thresholds (Fig. 3*J*), D_1_ cells had a higher threshold on average compared with non-D_1_ cells (Mann–Whitney *U* rank test, *p *=* *0.001). This difference was present even when restricting the set of non-D_1_ cells to those from sites where we found D_1_ cells (Mann–Whitney *U* rank test, *p *=* *0.001). We found no topographic organization according to intensity threshold for either population. We then compared the tuning bandwidth of cells from each population. Because traditional measures of bandwidth (e.g., BW10 and BW40) are challenging to estimate for high-threshold neurons, we first evaluated tuning bandwidth by pooling responses over the full range of intensities. We identified cells for which responses across sound frequencies were well fit by a Gaussian function (115 of 166 tone-responsive D_1_ neurons; and 79 of 104 tone-responsive non-D_1_ neurons), and used the width at half-maximum of this curve as an estimate of tuning bandwidth (Fig. 3*K*). We found no significant difference between the frequency-tuning bandwidth across these populations of neurons (Mann–Whitney *U* rank test, *p *=* *0.086). Similarly, we found no significant difference between cells with low enough intensity thresholds, which enable estimates of either BW10 (D_1_ cells, 97; and non-D_1_ cells, 70; Mann–Whitney *U* rank test, *p *=* *0.058) or BW40 (D_1_ cells, 18; non-D_1_, 24 cells; Mann–Whitney *U* rank test, *p *=* *0.809). Finally, we compared the proportion of low-threshold cells (<60 dB) with FRA shapes that matched each of the categories presented in Figure 3, *A–C* and *E–G*. We found no major differences between these proportions (Fig. 3*L*). Specifically, the ratio of neurons with a steep high-frequency slope was comparable between D_1_ and non-D_1_ neurons (D_1_, 22.7%; vs non-D_1_, 28.8%; Fisher’s exact test, *p *=* *0.574).

**Figure 4.**
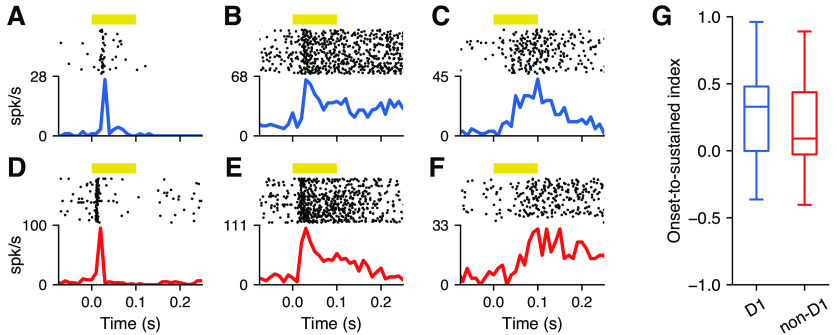
D_1_-expressing striatal neurons have similar response dynamics to neighboring neurons. ***A***, Example D_1_ neuron showing an onset response to pure tones. Yellow bar indicates the duration of the sound. Trials include all frequency–intensity combinations that evoked a response. ***B***, D_1_ neuron showing a sustained response that continues beyond the offset of the sound stimulus. ***C***, D_1_ neuron showing a delayed response. ***D–F***, Same as in ***A–C*** for non-D_1_ neurons. ***G***, The firing dynamics (a comparison between onset and sustained firing) for pure tones that elicited a response was similar between D_1_ and non-D_1_ neurons (Mann–Whitney *U* rank test, *p *=* *0.256).

We then evaluated whether the dynamics of the responses to pure tones differed between D_1_ and non-D_1_ neurons. For each cell, we focused on stimuli within the FRA and measured how firing changed over time after the stimulus onset. Some D_1_ neurons showed a brief response soon after the sound onset (Fig. 4*A*), other neurons showed sustained firing, sometimes extending beyond the end of the sound (Fig. 4*B*), while other neurons had a delayed response (Fig. 4*C*). We also found non-D_1_ neurons with each of these characteristics (Fig. 4*D–F*). On average, we found no difference between the latency of responses from the two populations of neurons (D_1_, 15.1 ms; non-D_1_, 14.4 ms; Mann–Whitney *U* rank test, *p *=* *0.656). To evaluate potential differences in response dynamics beyond the response latencies, we calculated an index that compares the magnitude of the onset response (0–50 ms) with the sustained part of the response (50–100 ms) for each neuron (Fig. 4*G*). We found no difference in this onset-to-sustained response index between the two populations (Mann–Whitney *U* rank test, *p *=* *0.256).

These results suggest that the frequency tuning of D_1_-expressing neurons in the posterior striatum is comparable with that of their neighboring neurons. We next wanted to evaluate the encoding of temporal acoustic features by these neuronal populations.

### D_1_-expressing neurons have responses to AM sounds comparable with those of their neighbors

**Figure 5.**
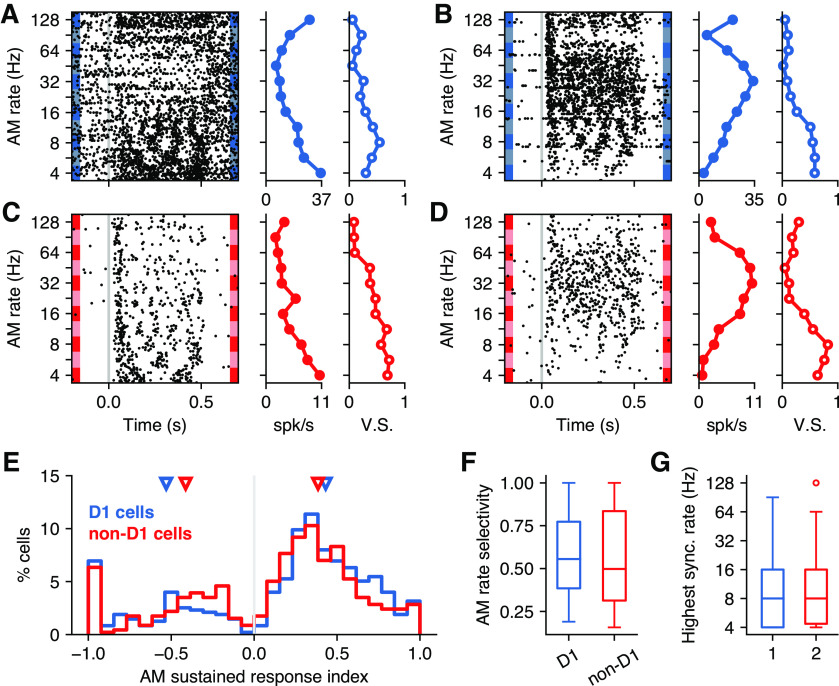
D_1_-expressing striatal neurons display similar selectivity to AM sounds compared with their neighbors. ***A***, Example responses of a D_1_ neuron to 500 ms AM white noise at different modulation rates. Left, Neural firing on each trial. Middle, Average firing during the sustained period for each AM rate. Right, Vector strength for each AM rate, representing how well spikes synchronize to the stimulus. ***B***, Responses to AM white noise for a different D_1_ neuron. This neuron is tuned to an intermediate modulation rate, but still shows the highest synchronization to low AM rates. ***C***, Responses of a non-D_1_ neuron showing a high level of synchronization to the stimulus and tuning to low AM rates. ***D***, Responses of a non-D_1_ neuron showing tuning to an intermediate modulation rate. ***E***, AM sound-evoked response index for the stimulus that elicited the largest response in each neuron of each class. Responses are calculated for the sustained portion of the response (100–500 ms). The triangles indicate the median response index calculated separately for neurons that responded by increasing or decreasing their firing. On average, D_1_ neurons showed larger responses than non-D_1_ neurons (Mann–Whitney *U* rank test, *p *=* *0.0013). ***F***, AM rate selectivity, calculated by comparing the strongest with the weakest sustained response across AM rates, was similar between D_1_ and non-D_1_ neurons (Mann–Whitney *U* rank test, *p *=* *0.578). ***G***, The highest AM rate that each neuron synchronized to was similar across cell classes (Mann–Whitney *U* rank test, *p *=* *0.832).

To test whether D_1_ neurons in the posterior striatum encoded temporal features of sounds with different fidelity compared with other neurons in this region, we evaluated the evoked responses of identified D_1_ and non-D_1_ cells to sinusoidally amplitude-modulated white noise at different modulation rates. A total of 475 D_1_ and 460 non-D_1_ neurons was recorded during the presentation of AM sounds. We found neurons from both classes that reliably responded to these sounds. As seen in neurons from other auditory regions, the responses of some cells were synchronized to the phase of the modulation, up to some modulation rate (Fig. 5*A–D*). While many neurons had the largest evoked firing for low modulation rates (Fig. 5*A*,*C*), other neurons were tuned to intermediate rates (Fig. 5*B*,*D*).

We calculated an SRI that compared the evoked response with baseline firing for each neuron and plotted this index for the modulation rate that elicited the most reliable evoked response in each neuron (Fig. 5*E*). For many neurons, the firing rate during the sustained portion was lower than the spontaneous firing (indicated by negative values of the index). Although the values of this index largely overlapped across the two populations of cells, we found that the strongest sound-evoked changes in firing for D_1_ neurons were larger than those for non-D_1_ neurons (median absolute index: D_1_, 0.47; vs non-D_1_, 0.39; Mann–Whitney *U* rank test, *p *=* *0.0013). The median values (Fig. 5*E*, triangles) were slightly larger for D_1_ neurons that had positive evoked responses and smaller for neurons with negative evoked responses, compared with median values for non-D_1_ neurons. This difference, however, disappeared when we restricted the analysis to the 193 non-D_1_ neurons recorded from sites where D_1_ cells were found (median: D_1_, 0.47; vs non-D_1_, 0.49; Mann–Whitney *U* rank test, *p *=* *0.212), suggesting that this result is influenced by the exact location of each recording site.

We next compared the modulation rate selectivity of cells from each population (Fig. 5*F*), focusing on the sustained period of the response. Given the rate modulation transfer function for each cell (Fig. 5*A–D*, middle panels), we calculated an AM rate selectivity index by comparing the maximum and minimum responses across modulation rates (including only neurons that were found to be responsive to AM sounds: D_1_ neurons, 81; non-D_1_ neurons, 52). We found no significant difference between the AM rate selectivity across these populations of neurons (Mann–Whitney *U* rank test, *p *=* *0.578). We also found that the most commonly preferred AM rate in both populations was the lowest rate tested (4 Hz: D_1_ cells, 37%; non-D_1_ cells, 42%) and found no difference in the distribution of preferred AM rate between D_1_ and non-D_1_ neurons (Mann–Whitney *U* rank test, *p *=* *0.744). Last, we tested whether neurons were organized topographically according to their AM rate preference, but found no correlation between the preferred rate and the neurons location for either D_1_ or non-D_1_ cells (D_1_: *r *<* *0.2, *p *>* *0.15 in all directions; non-D_1_: *r *<* *0.19, *p *>* *0.32 in all directions; Spearman’s correlation).

We then evaluated how well the evoked spikes synchronized to the modulation of the stimulus, using a vector strength metric on the sustained response for each cell (Fig. 5*A–D*, right panels). We found that responses from both populations synchronized more effectively to lower AM rates, and we found no difference in the rate that elicited the highest synchronization for each neuron across the two populations (Mann–Whitney *U* rank test, *p *=* *0.744). We also evaluated the highest modulation rate at which responses from each neuron synchronized with the stimulus, using the Rayleigh test for periodicity (Fig. 5*G*) and including only cells that synchronize to at least one AM rate (D_1_ neurons, 53; non-D_1_ neurons, 30). We found no difference in the highest synchronized modulation rate between the two populations (Mann–Whitney *U* rank test, *p *=* *0.832).

These results suggest that responses to amplitude modulated sounds by D_1_-expressing neurons in the posterior striatum are comparable with those of their neighboring neurons. Overall, the results above indicate that, in naive animals and outside the context of a task, different classes of neurons in the posterior tail of the striatum have access to and process acoustic features in a similar fashion.

## Discussion

In this study, we quantified the activity of neurons from the posterior tail of the striatum in response to sounds of different frequency or temporal structure. Taking advantage of optogenetic methods for identifying genetically distinct cell types during extracellular recordings, we compared the representation of sounds by distinct classes of striatal neurons. Specifically, we focused on one of the major cell classes in the posterior striatum, namely one that expresses the dopamine receptor D_1_ (and form the direct striatonigral pathway) and compared them to other neurons in the same striatal region. The cellular composition of the striatum is such that together D_1_-expressing and D_2_-expressing cells account for >90% of striatal neurons (Kreitzer and Malenka, 2008). While the proportions of these principal neurons in different subdivisions of the caudal striatum can vary greatly (Miyamoto et al., 2019), overall they are present in similar quantities. Therefore, it is likely that most of our recorded non-D_1_ neurons are in fact D_2_-expressing indirect pathway neurons. The possibility of observing differences in the representation of sounds by these cell classes was motivated by previous studies that suggest differential innervation of striatal pathways by cortical neurons (Lei et al., 2004; Kreitzer and Malenka, 2007; Wall et al., 2013). We found, however, that the representation of spectral and temporal features of sounds by D_1_-expressing neurons is similar to the representation of these features by neighboring neurons.

A common concern when applying methods for photo-identification during electrophysiological recordings is the possibility of observing laser-evoked responses from multisynaptic indirect activation by other neurons. This concern is less relevant when identifying striatal D_1_-expressing neurons as they are GABAergic in nature (and therefore their activation will result in the inhibition of their synaptic partners) and minimized by using only the early portion of the laser-evoked responses for identification (before recurrence can have a major impact). A more challenging limitation of these photo-identification methods results from possible changes in the spike shape if the photostimulation generates artificially large currents. Neurons in which this happens cannot be correctly identified by our algorithm and instead would be misclassified as D_1_ cells that do not respond to sounds and non-D_1_ cells suppressed by the laser. Visual inspection of the spike shapes and responses to laser stimulation across all cells suggests that these events were unlikely in our dataset.

Our results indicating that sound-responsive D_1_ neurons encode acoustic features with similar fidelity to those of sound-responsive non-D_1_ neurons was supported by multiple measurements, and it was robust to applying stricter criteria for inclusion of cells (e.g., using only cells with high firing rates to increase the reliability of response estimates; data not shown). Notably, comparisons across cell classes yielded slightly different results when we included all identified neurons or included only neurons recorded on the same electrodes. These observations cast doubt on the validity of differences observed across cell classes, as these effects could be explained by distinct levels of responsiveness by cells from different recorded locations. Variability in the recording locations across mice, together with the limited precision of our method for estimating the location of each recording make it impractical to derive further conclusions from our data regarding these potential differences. The one robust difference observed between the two cell populations was in their intensity threshold when presented with pure tones. In naive mice, we found that a larger proportion of D_1_ cells had high thresholds compared with non-D_1_ cells, although the responses evoked by the best tone stimuli for each neuron where comparable across the two populations. Whether this balance in thresholds changes as animals learn auditory tasks that result in striatal synaptic changes remains unknown.

Previous studies have observed strong selectivity to sound frequency in the responses of posterior striatal neurons of mice trained to perform auditory tasks (Guo et al., 2018; Chen et al., 2019). Our study complements these observations by demonstrating that these neurons display robust responses to sounds even in naive mice. Moreover, our study illustrates that the responses of posterior striatal neurons can be synchronized to the amplitude modulation of the stimulus or be tuned to specific modulation rates, as observed in auditory thalamic and cortical neurons (Ponvert and Jaramillo, 2019). One of these earlier studies evaluated the evoked responses to pure tones from different classes of striatal neurons identified according to their spike shapes (Chen et al., 2019). They found that subsets of neurons from all identified classes (medium spiny neurons, cholinergic interneurons, and fast-spiking interneurons) displayed responses to tones with various dynamics and frequency tuning. Because the group of cells classified as medium spiny neurons contained both direct and indirect pathway neurons, their study could not derive conclusions regarding potential differences between cells from these two pathways. Our study complements these results by demonstrating that direct pathway neurons encode sounds in a similar fashion to their neighbors. Because an overwhelming majority of neurons in the striatum consists of projection medium spiny cells, split evenly between the two striatal output pathways (Kreitzer and Malenka, 2008), it is likely that this conclusion extends to the comparison between direct versus indirect pathway neurons.

Little is known about potential anatomic differences in the auditory projections to different striatal neuron classes. However, a previous study found differences in the innervation from nonauditory cortical areas to D_1_- versus D_2_-expressing neurons in the dorsal striatum (Wall et al., 2013). In that case, somatosensory cortex preferentially innervated D_1_ neurons, while motor cortex preferentially innervated D_2_ neurons. In contrast, that same study found that thalamostriatal projections were balanced between D_1_ and D_2_ targets. These results suggest that even if there is a preference in auditory cortical projections toward one striatal cell class, thalamic neurons could provide balanced information to D_1_ and non-D_1_ cells, as observed in our study. Moreover, because corticostriatal neurons differ in their representation of AM sounds compared with thalamostriatal neurons (Ponvert and Jaramillo, 2019), one would expect these differences to be reflected in the responses of D_1_ and non-D_1_ neurons if cortical neurons preferentially target one particular class. Our results suggest that this is not the case, and that both D_1_ and non-D_1_ neurons have access to similar cortical-like representations of AM sounds (e.g., a preference to intermediate AM rates in some neurons).

**Figure 6.**
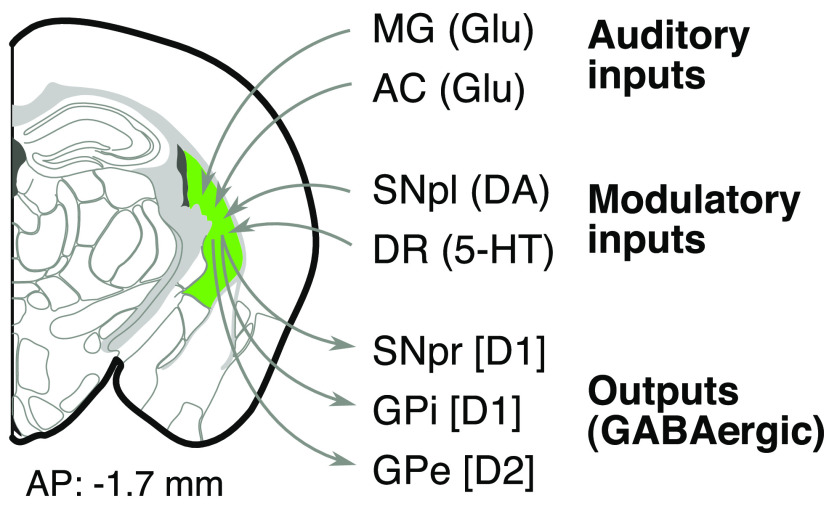
Inputs and outputs of the posterior tail of the striatum. The tail of the striatum (green) receives auditory glutamatergic inputs from the medial geniculate (MG) nucleus of the thalamus as well as primary and nonprimary fields of the auditory cortex (AC). It also receives dopaminergic (DA) inputs from the substantia nigra pars lateralis (SNpl) and serotonergic (5-HT) inputs from the dorsal raphe nucleus (DR). GABAergic medium spiny neurons that express either the dopamine receptor D_1_ or the dopamine receptor D_2_ form the main outputs of the tail of the striatum. D_1_ neurons target the substantia nigra pars reticulata (SNpr) and the internal globus pallidus (GPi). D_2_ neurons target the external globus pallidus (GPe). Based on the study by Valjent and Gangarossa (2021) and the Allen Mouse Brain Connectivity Atlas (Oh et al., 2014).

Our measurements also suggest a topographic organization of striatal neurons according to frequency preference. This observation, however, is at odds with results from tracing corticostriatal projections from regions of the primary auditory cortex tuned to difference sound frequencies in the mouse (Ghosh and Zador, 2021). While the tracing study found that fibers from the low-frequency region of A1 terminated more medially than those from the high-frequency region, we found that neurons located in the medial portions of the tail of the striatum preferred higher frequencies. These discrepancies could be explained by the fact that the tail of the striatum receives projections not only from A1, but also from the auditory thalamus and from secondary areas of the auditory cortex (Fig. 6), which may provide frequency information organized in a different way. However, it is puzzling why projections from different regions would not be topographically aligned. Because our results come from pooling neurons from multiple anteroposterior locations and from different mice, it may be necessary to further validate these results by performing high-density recordings across the medial–lateral axis from a single mouse.

Neurons in the posterior tail of the striatum of mice are necessary for sound-driven tasks such as those that require associating a sound with a reward port (Guo et al., 2018). Moreover, the activation of D_1_-expressing neurons in this region can consistently bias choices during these tasks without producing overt movements outside the task, in contrast to the movements generated by the activation of D_1_ neurons in other striatal regions (Guo et al., 2018). This suggest that posterior striatal neurons play a role in sound-driven decisions beyond simply promoting or inhibiting movement. In addition, these neurons receive dopaminergic and serotonergic neuromodulatory inputs (Fig. 6), suggesting that they integrate sensory information with reward and other task-related signals. Our results show that both neuron classes that form the output pathways from the posterior striatum have access to detailed spectrotemporal acoustic features and could therefore potentially influence behavioral responses to sounds. As D_1_- and D_2_-expressing cells send their outputs to distinct regions of the basal ganglia (Fig. 6), which in turn have different effects on movements and choices, it seems likely that the nervous system differentially adjusts the connections onto these pathways depending on the task at hand to implement distinct behavioral responses to different sounds. However, it remains unknown whether nonacoustic features that influence the activity of posterior striatal neurons, such as reward expectation and choice-related variables (Guo et al., 2019), are also represented similarly across cell classes, and whether differences in the representation of sounds emerge when investigating subgroups of neurons within each class (Gokce et al., 2016).

## Synthesis

Reviewing Editor: Christine Portfors, Washington State University Decisions are customarily a result of the Reviewing Editor and the peer reviewers coming together and discussing their recommendations until a consensus is reached. When revisions are invited, a fact-based synthesis statement explaining their decision and outlining what is needed to prepare a revision will be listed below. The following reviewer(s) agreed to reveal their identity: Catalina Valdés-Baizabal, Munenori ONO.

The reviewers agree that this manuscript contributes important information for understanding neuronal mechanisms for sound-driven animal behavior. This is an important first study of a region that is not well studied for its influence on auditory processing. The reviewers agree however that the manuscript would benefit from a more in-depth analysis of neuronal response properties that are common in the literature and that will help better characterize the types of neuronal responses in the tail of the striatum (FRA shape, BF, CF, threshold etc). It would also be helpful to include a more thorough discussion and a diagram that represents how the striatal tail receives dopaminergic (and non-dopaminergic) innervation (including from the cortex, thalamus and other structures, if any) in order to understand how the circuit is structured.

Reviewer 1

First, I want to congratulate the authors for their work, since recording awake animals is a difficult task.

I believe that the material and methods do not specify that handling sessions have been carried out on the animals prior to the electrophysiological recordings, in order to adapt them. Could you specify if you did?

Figure 3C shows how the firing rate decreases in non-D1 neurons (specify the n of this subgroup), but a clear increase in spontaneous activity is also seen. Discuss if necessary. If there are also cases in D1 neurons in which the firing rate evoked by sound decreases, give an example. It would be convenient to use the same scale of spikes per second in the current figure 3B and 3C, as well as to use the same scale (spikes per second) for 3A and its corresponding case of the subgroup in which the firing rate decreases.

In the discussion, a more in-depth explanation of why direct and indirect pathway neurons have similar responses to auditory stimuli is missing. I would like you to discuss in terms of hypotheses how cells would behave when dopamine is released and how auditory coding is expected to be modulated, specifying the differences that would then be found between both cell types.

It would be interesting to add a diagram that represents how the striatal tail receives dopaminergic (and non-dopaminergic) innervation (including from the cortex, thalamus and other structures, if any) in order to understand how the circuit is structured.

Perhaps not for this work, but taking into account the number of neurons recorded, they could characterize the types of responses of both D1 neurons and their neighbours (probably D2 neurons). There are works in which a classification of neurons has been characterized by the shape of the frequency response area. It might be interesting to see if there are differences between D1 neurons and their neighbours (probably D2 neurons) by looking beyond bandwidth.

Reviewer 2:

In this study, the authors examined the response properties of different classes of striatum neurons to sound in the awake mice, by the multichannel unit recordings combined with optogenetics photo-tagging technique. They found that both D1-positive and D1-negative neurons responded to tones and AM sound. Further, they elucidated that the neighboring neurons had similar frequency tuning properties. Recent studies have suggested that the striatum is a critical structure for sound-driven decision making, However the sound response properties of striatum neurons have many unknown aspects. This study is the first work that showed the response properties of different classes of striatum neurons to sound, so would contribute much to understanding the neuronal mechanism for sound -driven animal behaviors. The weak point of the present version is that the sound responses were not fully analyzed in detail, though authors recorded the responses from a substantial number of neurons. I believe that more intensive analysis on sound responses would make this study more valuable.

Major

1. More parameters should be measured from the responses to tones and compared between the classes of neurons; threshold, characteristic frequency, best frequency, 10dB bandwidth, 40dB bandwidth, first spike latency etc. (e.g. Egorova et al., 2001, 2020; Higgins et al., 2008). In the centers of auditory pathway, the neurons are known to have different shapes of frequency response areas (FRA). Please check the shapes of FRA and classify them. To analyze the properties of the response s to AM, the rate- and temporal- Modulation-transfer functions (e.g. Joris et al., 2001) should be measured. These functions may be classified into several shapes. Also, best rate- and temporal- modulation frequency should be measured.

These parameters should be compared between the classes of neurons.

2. In the auditory centers, it is known that there is spatial arrangement of processing the specific sound features. The most famous one is the frequency tonotopy. Further, in some auditory centers, some sound responses properties have spatial arrangement (e.g. dorsomedial arrangement of the first spike latency in the inferior colliculus; Schreiner&Lagner, 1988). Please check if there are some spatial arrangements relevant to specific parameters of sound feature (frequency, AM frequency, etc) or sound responses (latency ,threshold ,etc) in the striatum.

Minor

Line 140-142. In figure1C, D, the duration of the laser seems to be 100 ms. Why did you use only the responses during first 50ms? And how often and how many times was the laser stimulation given? The optogenetics stimulation sometimes may harm the neurons because of the large currents flow. Did the neurons state (e.g. spontaneous siring rate) not change after the lase stimuli?

Line 198-201. The shapes of the spikes evoked by the optogenetics stimuli might be different those of the naturally driven spikes because the artificial large current through ChR2 could interfere the size and time course of the action potential. Was the neuron which generated the laser-evoked spikes stably identified among the several neurons recorded by a tetrode?

Line 210-213. The authors found there were sound- sensitive and insensitive neurons in the striatum. Was the location of the recorded neuron relevant to the sound-sensitivity of the neuron? In striatum, did some region contain more sound- sensitive neurons than other region?

Line 255-258. Authors should describe the recording procedure for measuring the onset-to-sustained responses in the method. What frequency and intensity of the tone were used? How many times the stimulus was repeated? Also if reliable PSTH could be made, the time course of the PSTH should be evaluated and classified.

Line343-344. The results suggested that the D1-positive and D1-negative (putative D2-positive) neurons shared the similar frequency response properties in the local circuit of the striatum. Is there any histological evidence which support the similarity of the sound response properties of the neurons in the local circuit? Is there any tonotopically arranged projections from auditory structures to striatum?

Line348-350. Can the authors make some hypothesis how the similarity of the frequency response properties of D1- and D2- positive neurons in the local circuit contribute to the sound processing function of striatum (e.g. sound driven-decision making)?
